# Genomic epidemiology of *Candida auris* in a general hospital in Shenyang, China: a three-year surveillance study

**DOI:** 10.1080/22221751.2021.1934557

**Published:** 2021-06-06

**Authors:** Sufei Tian, Jian Bing, Yunzhuo Chu, Jingjing Chen, Shitong Cheng, Qihui Wang, Jingping Zhang, Xiaochun Ma, Baosen Zhou, Ling Liu, Guanghua Huang, Hong Shang

**Affiliations:** a National Clinical Research Center for Laboratory Medicine & Department of Laboratory Medicine, the First Hospital of China Medical University, Shenyang, People’s Republic of China; b State Key Laboratory of Genetic Engineering, School of Life Sciences, Fudan University, Shanghai, People’s Republic of China; c Department of Infectious Diseases & Department of Nosocomial Infection Control, the First Hospital of China Medical University, Shenyang, People’s Republic of China; d Department of critical care medicine, the First Hospital of China Medical University, Shenyang, People’s Republic of China; e Department of Clinical Epidemiology and Center of Evidence-Based Medicine, the First Hospital of China Medical University, Shenyang, People’s Republic of China; f State Key Laboratory of Mycology, Institute of Microbiology, Chinese Academy of Sciences, Beijing, People’s Republic of China

**Keywords:** *Candida auris*, whole-genome resequencing, South African clade, epidemiology, ERG11

## Abstract

*Candida auris* is an emerging pathogenic fungal species found worldwide. Since April 2016, *C. auris* colonization/infection cases have been found in a general hospital in Shenyang, China. The genome-based phylogenetic studies of these isolates remain undefined. In the current study, the microbiological characteristics and antifungal susceptibility of these *C. auris* isolates, which were collected in Shenyang during the three-year period (2016–2018), were investigated. Whole-genome sequencing was applied to investigate the genetic variation and molecular epidemiological characteristics. A total of 93 *C. auris* isolates, including 92 clinical isolates and 1 environmental screening isolate were identified. Among the investigated wards, the *C. auris* cases were the most prevalent (97.4%, 37/38) in four intensive care units (ICUs). The Shenyang isolates carrying the VF125AL mutation in the key drug-resistance gene *ERG11* were mainly fluconazole resistant and formed a distinct subclade under the South African clade according to the phylogenetic and population structural analyses. In addition, the Shenyang subclade was found to be closely related to the British subclade in the aspect of genetic distance. As a conclusion, this study provides an important clue for revealing the origin of *C. auris* found in Shenyang and could also contribute to improve the understanding of the epidemiological characteristics of *C. auris* worldwide.

## Introduction

*Candida auris* is a new pathogenic fungus that has elicited global concern due to its threat to public health [1,2]. It tends to cause outbreaks of nosocomial infections because it can colonize the skin or medical devices [3,4].

*C. auris*, which has significant geographical features, can be divided into five clades according to its genome sequence [5–7]. South Asian and South American clades mainly cause bloodstream infections (47%–76%), while the South African clade is responsible for a high percentage of urinary tract infections/colonization (38%).

To date, 38 cases of *C. auris* have been reported in China. In 2018, Wang et al. reported the first case of a non-drug-resistant *C. auris* strain (BJCA001) at Peking University People’s Hospital (Beijing) [8]. Almost concurrently, we reported the first 15 cases of fluconazole-resistant *C. auris* infections/colonization in Shenyang, China [9]. Subsequently, Chen et al. reported another two cases of fluconazole-resistant *C. auris* infection in Beijing [10]. Recently, Tse et al. reported 19 *C. auris* isolates in Hong Kong [11], and Tang et al. reported one *C. auris* isolate in Taiwan [12]. According to internal transcribed spacer (ITS) sequencing results, strain BJCA001 belongs to the South Asian clade, and the other 17 cases from mainland China all belong to the South African clade. To date, there have been no follow-up reports on the three cases of *C. auris* infection in Beijing, while more *C. auris-*positive cases appeared continuously in Shenyang (Supplementary Figure 1). However, the genome-based phylogenetic studies of these isolates remain undefined. In this study, we investigated the genetic features of *C. auris* in Shenyang using molecular epidemiological methods.

## Materials and methods

### The source of *C. auris* isolates

From April 2016 to November 2017, 35 isolates misidentified as “*Candida haemulonii*” were identified in the First Hospital of China Medical University (Shenyang) by the VITEK2 system and 15 of which were confirmed as *C. auris* using ITS sequencing [9]. From November 2017 to December 2018, *C. auris* cases were identified using the MALDI-TOF, VITEK-MS system with the self-established proteomic RUO database. Additionally, during the environmental screening process, one strain of *C. auris* was isolated from the bedrails of a *C. auris*-positive patient (RICU9) on March 9, 2018. After the isolates of the same specimen on the same day were removed, thus totally of 93 isolates, including 92 clinical isolates and 1 environmental screening isolate, were included in the subsequent analyses, such as drug sensitivity detection and genome sequencing analyses.

### Drug susceptibility testing

Antifungal susceptibility testing was performed using a commercial chromogenic susceptibility plate (Sensititre YeastOne, Thermo Fisher Scientific). For the anitifungal drugs, we used either the tentative MIC breakpoints published by the CDC (https://www.cdc.gov/fungal/candida-auris/c-auris-antifungal.html) or the derivative epidemiological cut-off values (ECVs) for *C. auris*, which was applied to categorize isolates as wild-type (WT) or non-WT (harbouring mechanisms of resistance) according to other published studies [13,14].

### Whole-genome resequencing

Whole-genome sequencing (WGS) was performed using the Illumina NovaSeq platform (by Berry Genomics Co., Beijing, China). Yeast cells of *C. auris* grown on YPD medium at 37°C for 16 h were used for genomic DNA extraction. The genome DNA of each isolate was extracted using a standard zymolyase protocol. For all 93 *C. auris* isolates, a paired-end library with an average insert size of 300 bp was prepared and sequenced using the Illumina NovaSeq platform with 2 × 150-bp reads. In addition, for one isolate (RICU_A1), single-molecule real-time (SMRT) sequencing was performed at Beijing Novogene Bioinformatics Technology Co., Ltd. (Beijing, China) using the PacBio RS II SMRT DNA sequencing system (Pacific Biosciences, Menlo Park, CA, USA). Specifically, 20-kb libraries were generated with the SMRT bell TM Template Prep Kit 1.0 (Pacific Biosciences). The sequence data from the Illumina platform were used to proofread the PacBio assembly sequence.

### Genome assembly

The Hierarchical Genome Assembly Process (HGAP) was used to assemble sequenced genomes. De novo assembly of the PacBio read sequences was carried out using continuous long reads (CLR), followed by the HGAP workflow (PacBioDevNet; Pacific Biosciences) as available in SMRT Analysis v2.3. HGAP consists of preassembly, de novo assembly with the Celera Assembler (CA), and assembly polishing with Quiver. CA software version 7.0 was utilized in the pre-assembly step, and the PacBioRs_PreAssembler with one module and a default minimum subread length of 500 bp, minimum read quality of 0.80, and minimum subread length of 7500 bp was used for error correction of the raw data generated using the PacBio RS II platform.

### Reference-based alignment and variant calling

Variant calling was performed for a total of 382 *C. auris* samples, including Shenyang (n=93), Beijing (n=1), CBS (n=4), and NCBI (n=284), using the GATK and SAMTools pipeline.

Raw reads were trimmed to remove low-quality (phred score ≤10), ambiguous, and adaptor bases using the FASTX-Toolkit v0.0.14 (http://hannonlab.cshl.edu/fastx_toolkit/index.html). The obtained clean reads were mapped to the RICU1_A1 genome using Bwa 0.7.17 [15] with default settings. SAMTools v1.361 [16] was employed to convert the alignment results into BAM format, and Picard Tools v1.56 (http://picard.source-forge.net) was used to remove duplicated sequences. The SAMTools and Genome Analysis Toolkit (GATK v2.7.2) [17] programmes were used to detect variable sites. For GATK, HaplotypeCaller was used, and the ploidy was set to 1. The parameters “stand_call_conf” (thresholds for low- and high-quality variation loci) and stand_emit_conf (minimum phred-scaled confidence threshold) were set to 50.0 and 20.0, respectively. The extracted high-quality SNPs were the consistent variation sites obtained from SAMTools and GATK. The variation sites with a coverage depth ≥20 were retained for subsequent analyses and final SNP extraction. For 93 *C. auris* isolates from Shenyang, we analyzed the amino acid substitutions and termination mutations among all isolates.

### Copy number variation analysis

The aforementioned duplication-removed BAM datasets were used to call copy number variation (CNVs) for each isolate. Genomic regions with CNVs were identified with the Splint script to avoid the “smiley pattern” bias [18]. A read depth of 1000-bp nonoverlapping windows across the genome was generated with default internal parameters. The relative copy numbers for each window were normalized using the median value of 1000-bp non-overlapping windows and visualized using R script. The predicted copy number of each chromosome was determined based on the mean read depth coverage in a given chromosome and normalized to the whole genome coverage. The gene copy numbers were calculated based on the average sequence depth across the gene region using SAMTools [15] and python script, and they were normalized to the average depth of all genes.

### Phylogeny, population structure, and genetic diversity

Population genomic analyses, including phylogeny, population structure, and genetic diversity, were performed for all isolates or sub-clades. Phylogenetic trees were constructed based on genome-wide SNPs using MrBayes v3.2 [19] (10 million generations) and RAxML v8.1.6 [20] (1000 bootstraps) with the General Time Reversible (GTR) model of nucleotide substitution and γ-distributed rates. The population structure was inferred using ADMIXTURE v1.2333 [21], and the best-fit K value was determined by the cross-validation (CV) procedure. The nucleotide diversity (π, average number of nucleotide differences per site) of each population was calculated using Variscan v2.0.639 [22].

### Phylogeographic reconstruction, substitution rates, and dynamics

Phylogeographic reconstruction and substitution rates were estimated using BEAST v1.8.010 [23], with a continuous time Markov Chain (CTMC) over discrete sampling locations. The Bayesian Markov chain Monte Carlo analysis was run for 100 million steps, 10% of which were removed as burn-in and sampled every 10,000 steps. Bayes factor tests were performed to provide statistical support for potential transmission routes between different geographic locations using SPREAD3 v1.0.616. [24]. The population dynamics of Shenyang *C. auris* were estimated using a flexible non-parametric Bayesian skyride model [25] incorporated in BEAST v1.8.0, with the HKY +γ model and a strict molecular clock.

## Results

### Spatial and temporal distribution characteristics of *C. auris* cases

**Figure 1.**
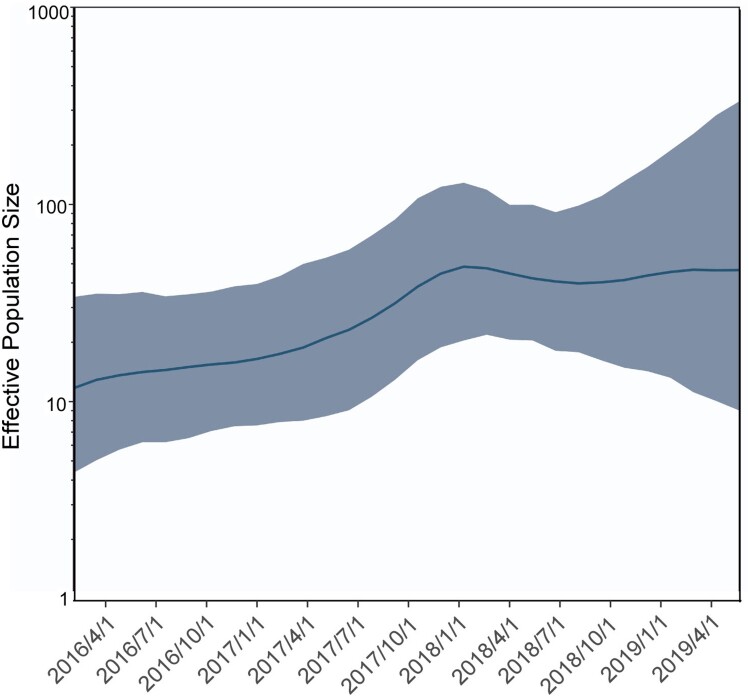
Temporal distribution of the effective population size for *C. auris* isolation from Shenyang. Gaussian Markov random field Bayesian skyride reconstruction of *C. auris*. The smooth blue line shows the effective population size, and the shadow shows the 95% highest posterior density.

A total of 38 patients were positive for *C. auris* from April 4, 2016 to December 2018. The 38 patients were from five wards. The most prevalent location with 19 cases was the Respiratory Intensive Care Unit (RICU), followed by 15 cases in the Neurosciences Intensive Care Unit (NICU). Only sporadic cases were found in the other three wards: two cases in the Surgery ICU (SICU), one case in the Neurosurgical Intensive Care Unit (NSICU), and one case in the Department of Respiratory Infection (RI). The case in the Department of RI had been previously admitted one time to the RICU in November 2016, and *C. auris* was detected in the patient's urine sample on November 7, 2018. The effective population size for *C. auris* isolation from Shenyang is presented in a temporal distribution in Figure 1.

### Clinical characteristics of *C. auris* isolates

The 93 strains were isolated from different specimen types, including 54 from urine, 18 from sputum, 8 from blood, 10 from catheters, and one each from stool, drainage fluid and other (bedrails). A total of 69 *C. auris* isolates were from single specimen types collected from 33 inpatients, i.e. such isolates were found only in one specimen type such as urine or sputum. Conversely, a total of 23 isolates were from multiple specimen types collected from 5 inpatients, i.e. such isolates were found in more than one specimen type (Table 1). For example, 9 isolates were collected from the same patient, RICU9, and the specimen types included blood (4), sputum (2), catheters (2), and stool (1).

**Table 1.** The source and distribution of the 93 *Candida auris*.

Case ID	Blood	Urine	Sputum	Catheter	Fluid	Stool	Other	Total
RICU1		1						1
RICU2		3						3
RICU3			1					1
RICU4	1		1	1(venous catheter)				3
RICU5				1(urinary catheter)				1
RICU6				1(urinary catheter)				1
RICU7		5		1(urinary catheter)				6
RICU8		2						2
RICU9	4		2	2(urinary catheter)		1		9
RICU10				1(tracheal catheter)				1
RICU11			7					7
RICU12			1					1
RICU13	1		2					3
RICU14				1(urinary catheter)				1
RICU15			2					2
RICU16			1	1(venous catheter)				2
RICU17	1							1
RICU18				1(urinary catheter)				1
RICU19		1						1
NICU2		1						1
NICU3		4						4
NICU4		2						2
NICU5		2						2
NICU6					1			1
NICU7		2						2
NICU8		5						5
NICU9		2						2
NICU10		1						1
NICU11	1							1
NICU12		1						1
NICU13		2						2
NICU14		1						1
NICU15		1						1
NICU16		4						4
NSICU1		2						2
SICU1		6						6
SICU2		4	1					5
RI		2						3
Environment							1	1
Total	8	54	18	10	1	1	1	93

Note: RICU Respiratory ICU; NICU Neurosciences ICU; NSICU Neurosurgical ICU; SICU Surgical ICU; RI Respiratory infection.

Among the 93 isolates, 34.4% (32/93) showed mixed growth of *C. auris* and other bacteria/fungi; thereinto, mixed growth of *C. auris* and one other kind of bacteria/fungi was observed for 24 isolates whereas mixed growth of *C. auris* and two other kinds of bacteria/fungi was observed for 8 isolates (Table 2). We found that 94.4% (17/18) of the sputum specimens had mixed growth. *C. auris* mostly grew with *Pseudomonas aeruginosa* (11 isolates) and *Acinetobacter baumannii* (6 isolates).

**Table 2.** The growth status of 93 *C. auris* strains isolated from different specimen types：*C. auris* only versus mixed growth of *C. auris* with bacteria/fungi.

Species	Urine	Sputum	Blood	Tracheal catheter	Fluid	Stool	Other	Total
*Candida auris* only	46	1	6	7		1		61
*Candida auris *+* Pseudomonas aeruginosa*	1	4		2				7
*Candida auris* + Pseudomonas aeruginosa + *Klebsiella pneumoniae*		1						1
*Candida auris *+* Pseudomonas aeruginosa *+ *Candida albicans*		3						3
*Candida auris* + *Acinetobacter baumannii*	1	3						4
*Candida auris* + *Acinetobacter baumannii *+ *Stenotrophomonas maltophilia*		1						1
*Candida auris* + *Acinetobacter baumannii+ Candida albicans*		1						1
*Candida auris* + *Klebsiella pneumoniae*	1							1
*Candida auris* + *Klebsiella pneumoniae + Stenotrophomonas maltophilia*		1						1
*Candida auris* + *Klebsiella pneumoniae *+ *Candida tropicalis*					1			1
*Candida auris *+* Chryseobacterium indologenes*	1							1
*Candida auris *+* Staphylococcus epidermidis*			1					1
*Candida auris *+* Staphylococcus haemolyticus*				1				1
*Candida auris *+ *Enterococcus faecium*	1		1					2
*Candida auris *+* Candida albicans*		1					1	2
*Candida auris* + *Candida tropicalis*	1							1
*Candida auris *+* Candida glabrata*	2							2
*Candida auris *+* Candida krusei*		1						1
*Candida auris *+* Aspergillus niger*		1						1
Total	54	18	8	10	1	1	1	93

All clinical specimens were inoculated on Sabouraud dextrose agar and CHROM agar Candida medium simultaneously. The microbiological characteristics of the colonies of *C. auris* were consistent with a previous description [9]. However, the mixed growth of *C. auris* and other bacteria and fungi was obviously slow, and the colonies were smaller. In such conditions, *C. auris* can be easily misdiagnosed, posing difficulties in terms of its detection.

### Characteristics of the antimicrobial susceptibility test (AST)

A total of 93 *C. auris* strains were subjected to AST, and the results are shown in Supplementary Table 1. Fluconazole MICs were ≥ 128 mg/L for all isolates, which were all above the ECV (non-WT). Voriconazole MICs were >1 mg/L for 86.0% (80/93) of the isolates. On the other hand, Itraconazole MICs were inhibited to as low as ≤0.25 mg/L for 93.5% (87/93) of the isolates. The resistance rate or non-WT was 2.2% (2/93) for echinocandin (Anidulafungin, Micafungin and Caspofungin) and the resistance rate or non-WT was 1.1% (1/93) for amphotericin B (Supplementary Table 2).

### Identification of mutations in the *ERG11* and *FKS1* genes

The VF125AL and I74L mutations located in the Erg11 amino acid sequence for all 93 *C. auris* highly resistant to fluconazole, as well as the S639F mutation located in the Fks1 amino acid sequence for two stains (RICU7_A33 and RICU7_A34) resistant to echinocandin, were detected via *C. auris* WGS (Supplementary Table 1). The sequence data for *ERG11* and *FKS1* were submitted to GenBank (accession number: MH124608 for isolate RICU4_A8 and MN088094 for isolate RICU7_A33 or RICU7_A34).

### Genome sequencing and worldwide phylogeny of *C. auris*

Ninety-three isolates were available for WGS: 47 from the RICU, 30 from the NICU, 11 from the SICU, two from the NSICU, two from the Department of RI, and one from environmental screening (C12_A109). BJCA001 from Beijing was also included in the analysis (Supplementary Table 1). For isolate RICU1_A1 (NCBI accession: ASM1421745v1), we developed a de novo assembly using PacBio reads, and the genome size was 12.38 Mb, similar to that of other *C. auris* strains (Supplementary Table 3). Phylogenetic analysis of all available *C. auris* genomes from Shenyang, China (n=93), Beijing, China (n=1), CBS (n=4), and NCBI (n=284) was performed using MrBayes and RaxML with 228,895 SNPs. Our phylogenetic and population structure analysis showed that all Shenyang isolates belonged to the South African *C. auris* clade (Figures 3 and 4). For the South African clade, 762 SNPs among the 168 isolates (93 from Shenyang, 10 from South Africa, and 65 from the UK) and 256 SNPs among 93 Shenyang isolates were detected. The 256 SNPs resulted in 80 amino acid substitution or termination mutations. The total SNP count for the Shenyang isolates was slightly greater than the value previously reported in the UK. All Shenyang isolates evolved into one large clade and seven sub-clades, as shown in Figure 3. The genetic variation of *C. auris* isolated from RICU was higher than that from other wards, and the genetic diversity of sub-clade 6 increased significantly.

### Phylogeography, mutation rates, and dynamic analysis

**Figure 2.**
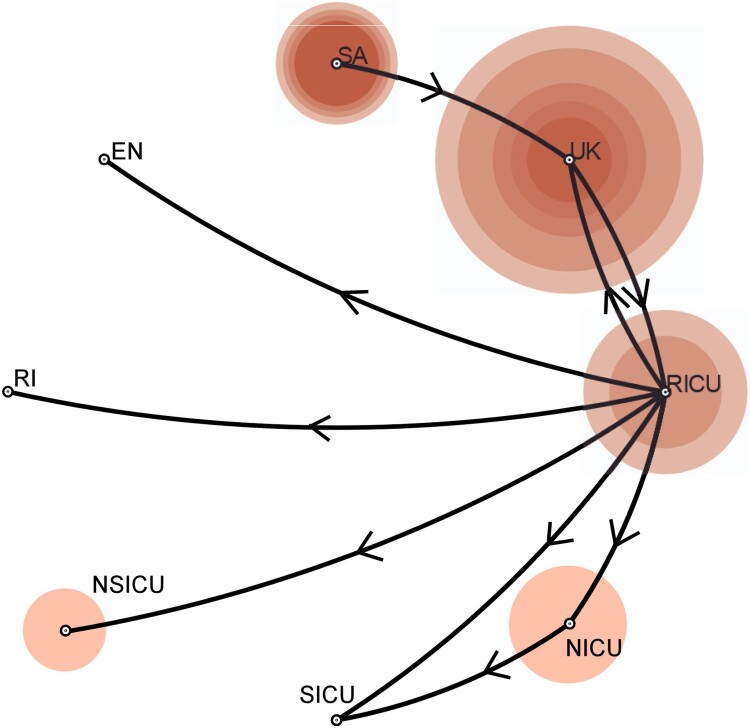
Reconstructed phylogeographic linkage, case counts, and effective population size of *C. auris* in Shenyang, China from 2016 to 2018. The phylogeographic linkage was constructed using BEAST and SpreadD3. Bayes factor tests were performed to provide statistical support for potential transmission routes between different geographic locations using SPREAD3. The phylogeographic linkage was constructed by routes with Bayes factor values >3. The size of the circle indicates the case counts of *C. auris*. Different offices are shown, including the RICU, NICU, NSICU, SICU, Department of RI, environment (EN), South Africa (SA), and the UK.

**Figure 3.**
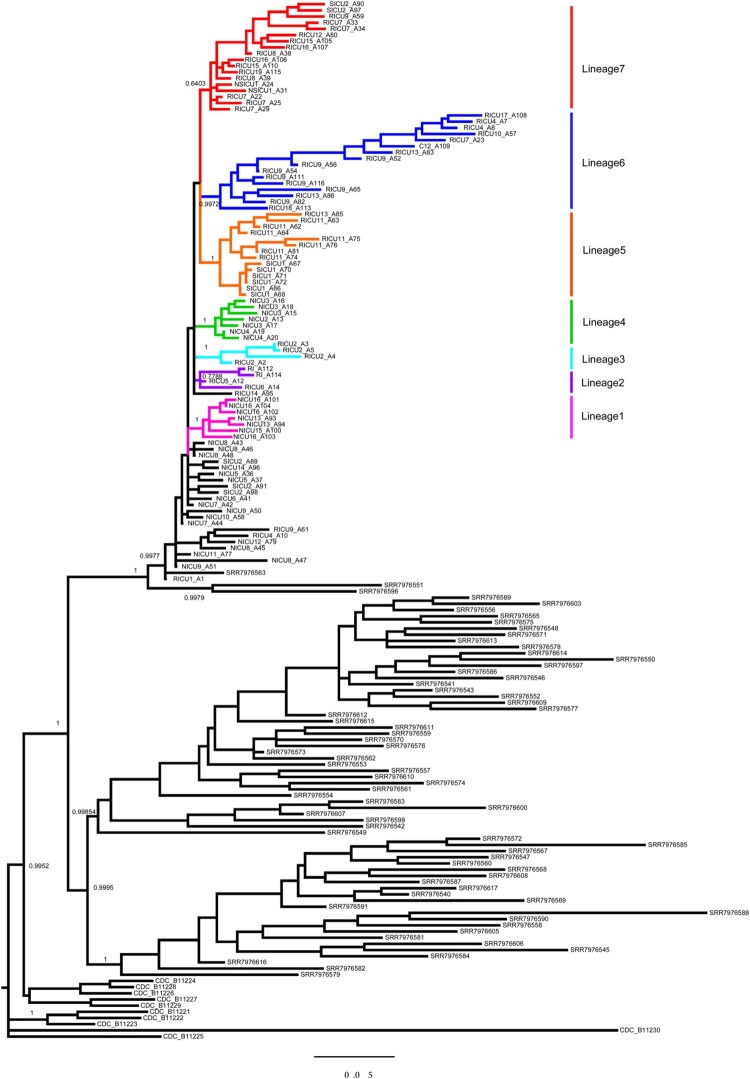
Bayesian phylogeny combined with *C. auris* from Shenyang and South Africa. A Bayesian phylogenetic tree was generated using MrBayes v3.2 (10 million generations) based on 762 SNPs. The GTR model of nucleotide substitution and γ-distributed rates among sites was adopted. Among a total of 168 samples, 93 were from Shenyang, 10 were from South Africa, and 65 were from the UK.

**Figure 4.**
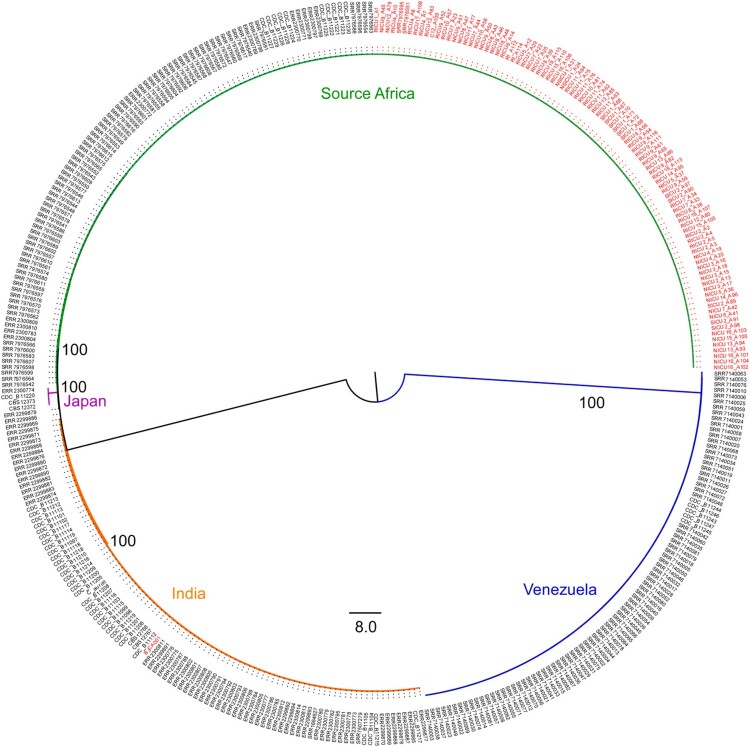
Maximum-likelihood phylogeny combined with a previously sequenced global collection. The tree was generated using the programme RAxML v7.3.2 with 228,895 SNPs. The GTR model, gamma distribution, and 500 bootstraps were adopted. Among a total of 382 samples, 93 were from Shenyang, one from Beijing, four from CBS, and 284 from NCBI. Different colored branches denote different lineages. The sample names from China are shown in red.

To explore the spatiotemporal relationships among different hospital departments, we performed a phylogeographic analysis using BEAST v1.8 and SPREAD3 v1.0.616. (Figures 2 and 3) using all 168 South African isolates. *C. auris* first appeared in the Department of RCCM (red), and the appearance time of *C. auris* in Shenyang was inferred to have occurred in the first half of 2014. It then spread from the RICU to the NICU, SICU, NSICU, Department of RI, and the environment. The secondary diffusion subsequently occurred from the NICU to the SICU. The first Shenyang *C. auris* isolate was putatively from the UK, and South African isolates were closer to the ancestor of the *C. auris* South African clade. The number of *C. auris* infection cases increased from 2016–2018. Their effective population size showed an increasing trend initially and then a slightly decreasing trend after our implementation of infection prevention and control measures against *C. auris* (Figure 1). The substitution rate for all 93 Shenyang *C. auris* was 6.56 mutations per genome per year (95% highest posterior density interval, 4.88–8.15) Figure 4.

## Discussion

The results of the present molecular epidemiological analysis showed that the Shenyang *C. auris* strains belonged to the South African clade, which are mainly fluconazole resistant and carry the VF125AL mutation in the key drug-resistance gene *ERG11* [6]. However, the correlation between resistance to echinocandin and the S639F mutation located in the Fks1 amino acid sequence has not yet been reported in South African strains (Supplementary Table 1 & DataSet S1). According to the results of the genetic analyses (DataSet S1), none of the genetic mutations or CNVs, which were found in the Shenyang strains, could possibly cause the MIC increase for voriconazole, itraconazole, and amphotericin B. Large region chromosome CNVs (DataSet S1) were observed in four strains (NICU12_A79, NICU16_A103, NICU3_A18, RICU8_A39), but no relative phenotypic variation was observed, necessitating further investigation of the function of CNVs. In terms of phylogeny, the Shenyang strains all originated from a single source and formed a distinct subclade. Cross-infection might exist among the RICU, NICU, SICU, NSICU, and Department of RI wards (Figure 2).

After analyzing the genomics of the Shenyang *C. auris* strains with the five major *C. auris* clades, the results showed that the genetic distance was closest between the South African clade (Shenyang strains) and the South Asian clade (Beijing BJCA001) and farthest between the South American clade and the East Asian clade [26]. The South African strain was the most ancient and later evolved into the British and Shenyang subclades; therefore, the genetic distance between the British and Shenyang subclades was very close. Moreover, one British *C. auris* strain (SRR7976563) even joined the Shenyang sub-clade, suggesting the possibility of genetic communication between these two subclades. However, neither the patients nor the doctors/nurses/staff had a history of travel to the UK, indicating that a more complex diffussion process might exist. In addition, the total number of SNPs in the Shenyang *C. auris* strains was slightly higher than that reported in the UK [3]. The genetic diversity (π) of different sub-clades (1–5, 7) ranged from 0·011–0·025, but that of sub-clade 6 had increased significantly (π=0·039). It is worth noting that sub-clade 6 included 62.5% of the candidemia-causing *C. auris* strains (Figure 3) and the strains isolated from multiple sites (RICU9_A52/A54/A56 from blood, RICU9_A65/A82 from sputum, RICU9_A111/A116 from a catheter tip, and C12-A109 from a related environment) of the patient (RICU9). The 22 *C. auris* strains were out of the 7 sub-clades, which might represent limited diffusion events for these strains. In addition, the South African *C. auris* clade is still evolving at a relatively constant rate, and the evolutionary positions of this clade deserve further investigation.

It is surprisingly that isolates of Shenyang were genetically related to South Africa clade, considering that Shenyang is far away from South Africa compared with Japan (East Asian clade) and India (South Asian clade). The underlying reasons require further study. Overall, at least two clades of *C. auris* (South Asian and South African clade) were found in China. The discovery of multiple *C. auris* clades in China may be explained by the continuously increasing global travelling and business exchanges in recent years, just as previously reported in the United States [27].

Last but not the least, we monitored isolates of the same specimen type (urine) collected from the same patient (RICU7) at different times, and we observed changes in drug resistance and genetic variation in these strains over time. Four strains collected before September 4, 2017, including RICU7_A22 (29/07/2017), RICU7_A23 (31/07/2017), RICU7_A25 (27/08/2017), and RICU7_A29 (04/09/2017), were sensitive to echinocandin. In contrast, the other two stains collected after September 4, 2017, including RICU7_A33 (13/09/2017) and RICU7_A34 (30/09/2017), were resistant to echinocandin. Moreover, mutation S639F in the *FKS1* gene was only found in these two strains. The patient had been treated with micafungin for 15 days (29/07/2017–12/08/2017) and for 6 days (01/09/2017–06/09/2017). This might suggest that early echinocin therapy was associated with echinocin resistance in *C. auris*.

It should also be noted that in this study, the tentative MIC breakpoints of echinomycin were set at 4 µg/mL for Micafungin/Anidulafungin and 2 µg/mL for Caspofungin, which was the most appropriate standard that we found for it was the most consistent with the results of the WGS sequence analysis for the drug resistance gene mutations.

In addition, 34.4% of *C. auris*-positive samples showed mixed growth of *C. auris* and other bacteria/fungi, which is higher than the 28% reported in South Korean studies [28]. Analyses of larger samples are needed in related research.

The *C. auris* strains identified in China (Shenyang) formed a distinct subclade under the South African clade. In addition, the Shenyang subclade was found to be closely related to the British subclade in the aspect of genetic distance. As a conclusion, this study provides an important clue for revealing the origin of *C. auris* found in Shenyang and could also contribute to improve the understanding of the epidemiological characteristics of *C. auris* worldwide.

## Supplementary Material

__1.jpg

Supplementary_table_3.docx

Supplementary_table_2.docx

Supplementary_table_1_.docx

## Data Availability

Pacbio and illumina reads and assembly from this study were deposited at DDBJ/ENA/GenBank under BioProject PRJNA549344. Other sequencing read data that can be found in BioProjects are PRJNA267757, PRJNA328792, PRJEB20230, PRJNA415955, PRJNA470683, and PRJEB9463.
