# Supplementary figures and images for: Genomic epidemiology of Candida auris in a general hospital in Shenyang, China: a three-year surveillance study
Source: Emerg Microbes Infect. 2021 Jun 6;10(1):1088–96. doi: 10.1080/22221751.2021.1934557 (PMC8183536; doi:10.1080/22221751.2021.1934557)

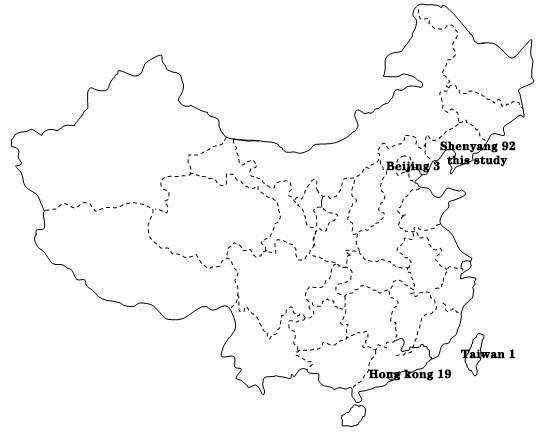

Supplement: __1.jpg [file TEMI_A_1934557_SM4732.jpg]
