# Supplementary material for: Genomic epidemiology of Candida auris in a general hospital in Shenyang, China: a three-year surveillance study
Source: Emerg Microbes Infect. 2021 Jun 6;10(1):1088–96. doi: 10.1080/22221751.2021.1934557 (PMC8183536; doi:10.1080/22221751.2021.1934557)
Supplement: Supplementary_table_3.docx [file TEMI_A_1934557_SM4731.docx]

**Supplementary Table 3 C*. auris* genome features**

| **Organism** | **Accession number** | **Size**  **(Mb)** | **No· of**  **scaffolds** | **N50**  **(Mb)** | **GC**  **(%)** | **NO· of**  **genes** | **Ave· gene**  **size (bp)** | **Ploidy** |
| --- | --- | --- | --- | --- | --- | --- | --- | --- |
| *Candida* *auris* **RICU1_A1** | **ASM1421745v1** | 12·4 | 7 | 2·37 | 46·5 | 5359 | 1,499 | haploid |
| *Candida* *auris B8441* | **GCA_002759435** | 12·4 | 15 | 1·1 | 45·2 | 5421 | 1,553 | haploid |
| *Candida albicans** | **GCA_000182965** | 14·3 | 9 | 2·23 | 33·5 | 6,107 | 1,468 | diploid |
| *Candida tropicalis** | **GCA_000006335** | 14·5 | 23 | 1·65 | 33·1 | 6,258 | 1,454 | diploid |
| *Candida* *parapsilosis** | **GCA_000182765** | 13·1 | 9 | 2·09 | 38·7 | 5,733 | 1,533 | diploid |
| *Lodderomyces* *elongisporus** | **GCA_000149685** | 15·4 | 27 | 2·01 | 37·0 | 5,802 | 1,530 | diploid |
| *Candida* *guilliermondii** | **GCA_000149425** | 10·6 | 9 | 1·70 | 43·8 | 5,920 | 1,402 | haploid |
| Clavispora *lusitaniae** | **GCA_000003835** | 12·1 | 9 | 1·87 | 44·5 | 5,941 | 1,382 | haploid |
| *Debaryomyces* *hanseni** | **GCA_000006445** | 12·2 | 7 | 2·01 | 36·3 | 6,318 | 1·,382 | haploid |
| *Yarrowia lipolytica* | **GCA_000002525** | 20·8 | 7 | 3·63 | 45·17 | 6,472 | 1,381 | haploid |

Mb, megabase pair; bp, base pair; NO., number; Ave., average; N50, defined as the sequence length of the shortest contig at 50% of the total genome length; ***** These species data come from Butler et al., 2009; Data of *Candida auris* B8441 and *Yarrowia lipolytica* come from the direct analysis of the public fasta and feature file of genome sequence, gene sequence on NCBI (https://www.ncbi. nlm.nih.gov/). The accession numbers for all assemblies were shown.
