# Supplementary material for: Genomic epidemiology of Candida auris in a general hospital in Shenyang, China: a three-year surveillance study
Source: Emerg Microbes Infect. 2021 Jun 6;10(1):1088–96. doi: 10.1080/22221751.2021.1934557 (PMC8183536; doi:10.1080/22221751.2021.1934557)
Supplement: Supplementary_table_2.docx [file TEMI_A_1934557_SM4730.docx]

Supplementary Table 2 Antifungal susceptibility data for 93 *Candida auris*

|  | MIC range, μg/mL | MIC_50_, μg/mL | MIC_90_, μg/mL | **GM**  **μg/mL** | **dECV**  **μg/mL** | **No.(%R or non-WT)** |
| --- | --- | --- | --- | --- | --- | --- |
| Fluconazole | 128->256 | >256 | >256 | **253** | **64** | **93 (100%)** |
| Voriconazole | 0.5-4 | 1 | 2 | **1.15** | **NA** | **NA** |
| Itraconazole | 0.06-4 | 0.25 | 0.25 | **0.21** | **NA** | **NA** |
| Amphotericin B | 0.5-2 | 1 | 1 | **0.89** | **≥2** | **1 (1.1%)** |
| Anidulafungin | 0.12-8 | 0.25 | 0.5 | **0.23** | **≥ 4** | **2 (2.2%)** |
| Micafungin | 0.06-8 | 0.12 | 0.12 | **0.12** | **≥ 4** | **2 (2.2%)** |
| Caspofungin | 0.06-4 | 0.12 | 0.25 | **0.18** | **≥ 2** | **2 (2.2%)** |
| Flucytosine | <0.06-0.25 | 0.12 | 0.12 | **0.11** | **NA** | **NA** |
| Posaconazole | 0.03-0.25 | 0.06 | 0.12 | **0.07** | **NA** | **NA** |

MIC, minimum inhibitory concentration; MIC50/90, MIC that inhibits 50% and 90% of the isolates, respectively; GM, geometric mean; dECV, derivative epidemiological cut-off value; R, resistant; non-WT, non-wild-type; NA, not applicable
