# Supplementary material for: Genomic epidemiology of Candida auris in a general hospital in Shenyang, China: a three-year surveillance study
Source: Emerg Microbes Infect. 2021 Jun 6;10(1):1088–96. doi: 10.1080/22221751.2021.1934557 (PMC8183536; doi:10.1080/22221751.2021.1934557)
Supplement: Supplementary_table_1_.docx [file TEMI_A_1934557_SM4729.docx]

Supplementary Table 1 Clinical details, in vitro antifungal susceptibility profile, **WGS accession number** and Amino acid substitutions in *ERG11* and *FKS1* in *Candida auris* isolates from **Shenyang(93)and Beijing(1)**

| **Patient （Sex /Age.y)** | **Isolate** | **Ward** | Source | Isolation date | **WGS accession number** | **Sub-clade** | MIC (minimal inhibitory concentration μg/ml) | | | | | | | | | Mutations in *ERG11* | Mutations in *FKS1* |
| --- | --- | --- | --- | --- | --- | --- | --- | --- | --- | --- | --- | --- | --- | --- | --- | --- | --- |
|  |  |  |  |  |  |  | AMB | AFG | MFG | CFG | 5-FC | POS | VRC | ITC | FLU |  |  |
| **Beijing（F/76）** | BJCA001 | - | Bronchoalveolar lavage fluid | - | **SRR9316737** | **-** | 0·25 | 0·12 | 0·06 | 0·06 | <0.06 | 0·02 | 0·02 | 0·03 | 2·00 | - | - |
| **RICU1（M/70）** | **RICU1_A1*** | RICU | urine | 06/04/2016 | **SRR9316788** | **other** | 0.5 | 0.12 | 0.06 | 0.06 | <0.06 | 0.03 | 0.5 | 0.06 | **256** | VF125AL (I74L) | - |
| **RICU2（F/69）** | **RICU2_A2*** | RICU | urine | 03/06/2016 | **SRR9316744** | **sub-clade3** | 0.5 | 0.12 | 0.12 | 0.12 | <0.06 | 0.06 | 1 | 0.12 | **256** | VF125AL (I74L) | - |
|  | RICU2_A4 | RICU | urine | 20/06/2016 | **SRR9316763** | **sub-clade3** | 1 | 0.12 | 0.12 | 0.12 | 0.06 | 0.06 | **4** | **1** | **>256** | VF125AL (I74L) | - |
|  | RICU2_A5 | RICU | urine | 01/07/2016 | **SRR9316769** | **sub-clade3** | **2** | 0.25 | 0.12 | 0.12 | 0.06 | 0.06 | 1 | 0.25 | **>256** | VF125AL (I74L) | - |
| **RICU3（F/69）** | **RICU3_A3*** | RICU | sputum | 17/06/2016 | **SRR9316754** | **sub-clade3** | 0.5 | 0.12 | 0.06 | 0.06 | <0.06 | 0.03 | 0.5 | 0.06 | **256** | VF125AL (I74L) | - |
| **RICU4（M/56）** | RICU4_A7 | RICU | sputum | 05/08/2016 | **SRR9316779** | **sub-clade6** | 1 | 0.25 | 0.12 | 0.25 | 0.25 | 0.12 | **4** | 0.5 | **>256** | VF125AL (I74L) | - |
|  | **RICU4_A8*** | RICU | blood | 10/09/2016 | **SRR9316801** | **sub-clade6** | 0.5 | 0.12 | 0.12 | 0.12 | <0.06 | 0.06 | 1 | 0.12 | **256** | VF125AL (I74L) | - |
|  | RICU4_A10 | RICU | venous catheter | 19/09/2016 | **SRR9316789** | **other** | 1 | 0.5 | 0.5 | 0.25 | 0.12 | 0.12 | 2 | 0.25 | **>256** | VF125AL (I74L) | - |
| **RICU5（F/82）** | **RICU5_A12*** | RICU | urinary catheter | 07/12/2016 | **SRR9316729** | **sub-clade2** | 0.5 | 0.12 | 0.12 | 0.12 | <0.06 | 0.03 | 0.5 | 0.06 | **256** | VF125AL (I74L) | - |
| **RICU6（M/70）** | **RICU6_A14*** | RICU | urinary catheter | 26/01/2017 | **SRR9316738** | **sub-clade2** | 0.5 | 0.12 | 0.12 | 0.12 | <0.06 | 0.03 | 0.5 | 0.12 | **128** | VF125AL (I74L) | - |
| **RICU7（F/63）** | **RICU7_A22*** | RICU | urine | 29/07/2017 | **SRR9316746** | **sub-clade7** | 0.5 | 0.12 | 0.12 | 0.12 | <0.06 | 0.06 | 1 | 0.12 | **256** | VF125AL (I74L) | - |
|  | RICU7_A23 | RICU | urinary catheter | 31/07/2017 | **SRR9316750** | **sub-clade6** | 1 | 0.25 | 0.12 | 0.25 | 0.12 | 0.06 | 1 | 0.25 | **>256** | VF125AL (I74L) | - |
|  | RICU7_A25 | RICU | urine | 27/08/2017 | **SRR9316748** | **sub-clade7** | 1 | 0.12 | 0.12 | 0.12 | 0.06 | 0.06 | 1 | 0.25 | **256** | VF125AL (I74L) | - |
|  | RICU7_A29 | RICU | urine | 04/09/2017 | **SRR9316749** | **sub-clade7** | 1 | 0.25 | 0.12 | 0.12 | 0.06 | 0.06 | 2 | 0.25 | **>256** | VF125AL (I74L) | - |
|  | RICU7_A33 | RICU | urine | 11/09/2017 | **SRR9316752** | **sub-clade7** | 1 | **8** | **8** | **4** | 0.12 | 0.06 | 1 | 0.25 | **>256** | VF125AL (I74L) | **S639F** |
|  | RICU7_A34 | RICU | urine | 13/09/2017 | **SRR9316753** | **sub-clade7** | 1 | **8** | **8** | **4** | 0.12 | 0.06 | 1 | 0.25 | **>256** | VF125AL (I74L) | **S639F** |
| **RICU8（F/73）** | **RICU8_A38*** | RICU | urine | 10/10/2017 | **SRR9316765** | **sub-clade7** | 0.5 | 0.12 | 0.06 | 0.06 | <0.06 | 0.03 | 0.5 | 0.06 | **256** | VF125AL (I74L) | - |
|  | RICU8_A39 | RICU | urine | 14/10/2017 | **SRR9316764** | **sub-clade7** | 1 | 0.25 | 0.12 | 0.25 | 0.12 | 0.06 | 1 | 0.25 | **>256** | VF125AL (I74L) | - |
| **RICU9（M/72）** | RICU9_A52 | RICU | blood | 31/12/2017 | **SRR9316772** | **sub-clade6** | 1 | 0.5 | 0.5 | 0.25 | 0.12 | 0.12 | 2 | 0.25 | **>256** | VF125AL (I74L) | - |
|  | RICU9_A54 | RICU | blood | 15/01/2018 | **SRR9316773** | **sub-clade6** | 1 | 0.25 | 0.12 | 0.12 | 0.12 | 0.06 | 1 | 0.25 | **256** | VF125AL (I74L) | - |
|  | RICU9_A56 | RICU | blood | 17/01/2018 | **SRR9316774** | **sub-clade6** | 1 | 0.25 | 0.12 | 0.25 | 0.12 | 0.06 | 1 | 0.25 | **256** | VF125AL (I74L) | - |
|  | RICU9_A59 | RICU | blood | 24/02/2018 | **SRR9316777** | **sub-clade7** | 1 | 0.25 | 0.12 | 0.25 | <0.06 | 0.06 | 1 | 0.25 | **256** | VF125AL (I74L) | - |
|  | RICU9_A61 | RICU | stool | 27/02/2018 | **SRR9316785** | **other** | 1 | 0.5 | 0.12 | 0.25 | 0.12 | 0.12 | 2 | 0.25 | **>256** | VF125AL (I74L) | - |
|  | RICU9_A65 | RICU | sputum | 28/03/2018 | **SRR9316781** | **sub-clade6** | 1 | 0.25 | 0.12 | 0.12 | 0.12 | 0.12 | 1 | 0.25 | **>256** | VF125AL (I74L) | - |
|  | RICU9_A82 | RICU | sputum | 28/05/2018 | **SRR9316811** | **sub-clade6** | 1 | 0.25 | 0.12 | 0.25 | 0.12 | 0.06 | 1 | 0.25 | **>256** | VF125AL (I74L) | - |
|  | RICU9_A111 | RICU | urinary catheter | 07/11/2018 | **SRR9316727** | **sub-clade6** | 0.5 | 0.25 | 0.06 | 0.25 | 0.12 | 0.12 | 1 | 0.25 | **256** | VF125AL (I74L) | - |
|  | RICU9_A116 | RICU | urinary catheter | 20/12/2018 | **SRR9316728** | **sub-clade6** | 1 | 0.25 | 0.12 | 0.25 | 0.12 | 0.12 | 2 | 0.25 | **>256** | VF125AL (I74L) | - |
| **RICU10（F/83）** | RICU10_A57 | RICU | tracheal catheter | 24/01/2018 | **SRR9316775** | **sub-clade6** | 1 | 0.25 | 0.12 | 0.25 | 0.12 | 0.12 | 2 | 0.25 | **>256** | VF125AL (I74L) | - |
| **RICU11（M/79）** | RICU11_A62 | RICU | sputum | 02/03/2018 | **SRR9316784** | **sub-clade5** | 1 | 0.25 | 0.12 | 0.25 | 0.12 | 0.06 | 1 | 0.25 | **>256** | VF125AL (I74L) | - |
|  | RICU11_A63 | RICU | sputum | 05/03/2018 | **SRR9316787** | **sub-clade5** | 1 | 0.25 | 0.12 | 0.25 | 0.12 | 0.06 | 1 | 0.25 | **256** | VF125AL (I74L) | - |
|  | RICU11_A64 | RICU | sputum | 26/03/2018 | **SRR9316786** | **sub-clade5** | 1 | 0.25 | 0.12 | 0.12 | 0.12 | 0.06 | 1 | 0.25 | **>256** | VF125AL (I74L) | - |
|  | RICU11_A74 | RICU | sputum | 23/04/2018 | **SRR9316804** | **sub-clade5** | 1 | 0.25 | 0.12 | 0.25 | 0.06 | 0.06 | 1 | 0.25 | **>256** | VF125AL (I74L) | - |
|  | RICU11_A75 | RICU | sputum | 28/04/2018 | **SRR9316805** | **sub-clade5** | 1 | 0.25 | 0.12 | 0.12 | 0.06 | 0.06 | 1 | 0.25 | **>256** | VF125AL (I74L) | - |
|  | RICU11_A76 | RICU | sputum | 11/05/2018 | **SRR9316802** | **sub-clade5** | 1 | 0.25 | 0.12 | 0.12 | 0.12 | 0.06 | 2 | 0.25 | **>256** | VF125AL (I74L) | - |
|  | RICU11_A81 | RICU | sputum | 23/05/2018 | **SRR9316799** | **sub-clade5** | 1 | 0.25 | 0.12 | 0.25 | 0.12 | 0.12 | 2 | 0.25 | **>256** | VF125AL (I74L) | - |
| **RICU12（M/81）** | RICU12_A80 | RICU | sputum | 15/05/2018 | **SRR9316798** | **sub-clade7** | 1 | 0.25 | 0.12 | 0.12 | 0.25 | 0.12 | 2 | 0.25 | **>256** | VF125AL (I74L) | - |
| **RICU13（F/74）** | RICU13_A83 | RICU | sputum | 31/05/2018 | **SRR9316810** | **sub-clade6** | 1 | 0.5 | 0.12 | 0.25 | 0.12 | 0.12 | 2 | 0.25 | **>256** | VF125AL (I74L) | - |
|  | RICU13_A85 | RICU | blood | 25/06/2018 | **SRR9316809** | **sub-clade5** | 1 | 0.5 | 0.12 | 0.25 | 0.12 | 0.12 | 2 | 0.25 | **>256** | VF125AL (I74L) | - |
|  | RICU13_A86 | RICU | sputum | 27/06/2018 | **SRR9316808** | **sub-clade6** | 1 | 0.12 | 0.06 | 0.12 | <0.06 | 0.03 | 0.5 | 0.12 | **256** | VF125AL (I74L) | - |
| **RICU14（M/89）** | RICU14_A95 | RICU | urinary catheter | 23/08/2018 | **SRR9316816** | **other** | 1 | 0.25 | 0.12 | 0.25 | <0.06 | 0.06 | 1 | 0.12 | **256** | VF125AL (I74L) | - |
| **RICU15（M/59）** | RICU15_A105 | RICU | sputum | 30/09/2018 | **SRR9316795** | **sub-clade7** | 1 | 0.12 | 0.06 | 0.12 | 0.12 | 0.12 | 1 | 0.25 | **256** | VF125AL (I74L) | - |
|  | RICU15_A110 | RICU | sputum | 11/10/2018 | **SRR9316726** | **sub-clade7** | 0.5 | 0.25 | 0.12 | 0.12 | <0.06 | 0.06 | 1 | 0.25 | **256** | VF125AL (I74L) | - |
| **RICU16（M/83）** | RICU16_A106 | RICU | sputum | 05/10/2018 | **SRR9316796** | **sub-clade7** | 1 | 0.12 | 0.06 | 0.12 | 0.12 | 0.06 | 1 | 0.25 | **256** | VF125AL (I74L) | - |
|  | RICU16_A107 | RICU | venous catheter | 08/10/2018 | **SRR9316797** | **sub-clade7** | 1 | 0.25 | 0.06 | 0.12 | 0.12 | 0.12 | 2 | 0.25 | **256** | VF125AL (I74L) | - |
| **RICU17（F/61）** | RICU17_A108 | RICU | blood | 14/12/2016 | **SRR9316724** | **sub-clade6** | 1 | 0.12 | 0.12 | 0.25 | 0.12 | 0.06 | 1 | 0.25 | **256** | VF125AL (I74L) | - |
| **RICU18（F/63）** | RICU18_A113 | RICU | urinary catheter | 13/11/2018 | **SRR9316721** | **sub-clade6** | 1 | 0.25 | 0.12 | 0.25 | 0.12 | 0.12 | 1 | 0.25 | **>256** | VF125AL (I74L) | - |
| **RICU19（M/78）** | RICU19_A115 | RICU | sputum | 20/11/2018 | **SRR9316723** | **sub-clade7** | 1 | 0.25 | 0.12 | 0.25 | 0.12 | 0.12 | 2 | 0.25 | **256** | VF125AL (I74L) | - |
| **NICU2（M/58）** | **NICU2_A13*** | NICU | urine | 24/12/2016 | **SRR9316739** | **sub-clade4** | 1 | 0.12 | 0.12 | 0.12 | <0.06 | 0.06 | 1 | 0.12 | **256** | VF125AL (I74L) | - |
| **NICU3（M/86）** | **NICU3_A15*** | NICU | urine | 04/02/2017 | **SRR9316741** | **sub-clade4** | 0.5 | 0.12 | 0.12 | 0.12 | <0.06 | 0.06 | 0.5 | 0.12 | **256** | VF125AL (I74L) | - |
|  | NICU3_A16 | NICU | urine | 08/02/2017 | **SRR9316740** | **sub-clade4** | 1 | 0.12 | 0.12 | 0.12 | <0.06 | 0.06 | 1 | 0.25 | **256** | VF125AL (I74L) | - |
|  | NICU3_A17 | NICU | urine | 21/02/2017 | **SRR9316743** | **sub-clade4** | 1 | 0.25 | 0.12 | 0.12 | <0.06 | 0.06 | 1 | 0.25 | **256** | VF125AL (I74L) | - |
|  | NICU3_A18 | NICU | urine | 27/02/2017 | **SRR9316742** | **sub-clade4** | 1 | 0.12 | 0.12 | 0.12 | <0.06 | 0.06 | 1 | 0.25 | **256** | VF125AL (I74L) | - |
| **NICU4（M/49）** | **NICU4_A19*** | NICU | urine | 17/03/2017 | **SRR9316745** | **sub-clade4** | 0.5 | 0.12 | 0.12 | 0.12 | <0.06 | 0.06 | 1 | 0.12 | **256** | VF125AL (I74L) | - |
|  | NICU4_A20 | NICU | urine | 31/03/2017 | **SRR9316747** | **sub-clade4** | 1 | 0.25 | 0.12 | 0.12 | 0.06 | 0.06 | **4** | 0.25 | **>256** | VF125AL (I74L) | - |
| **NICU5（F/86）** | **NICU5_A36*** | NICU | urine | 30/09/2017 | **SRR9316756** | **other** | 0.5 | 0.12 | 0.06 | 0.06 | <0.06 | 0.03 | 0.5 | 0.06 | **256** | VF125AL (I74L) | - |
|  | NICU5_A37 | NICU | urine | 07/10/2017 | **SRR9316757** | **other** | 1 | 0.25 | 0.12 | 0.12 | 0.06 | 0.06 | 1 | 0.25 | **>256** | VF125AL (I74L) | - |
| **NICU6（F/82）** | **NICU6_A41*** | NICU | fluid | 17/10/2017 | **SRR9316762** | **other** | 0.5 | 0.12 | 0.06 | 0.06 | <0.06 | 0.03 | 0.5 | 0.06 | **256** | VF125AL (I74L) | - |
| **NICU7（M/33）** | NICU7_A42 | NICU | urine | 22/11/2017 | **SRR9316761** | **other** | 1 | 0.25 | 0.12 | 0.12 | <0.06 | 0.06 | 1 | 0.25 | **256** | VF125AL (I74L) | - |
|  | NICU7_A44 | NICU | urine | 24/11/2017 | **SRR9316759** | **other** | 1 | 0.25 | 0.12 | 0.25 | 0.06 | 0.06 | 1 | 0.25 | **256** | VF125AL (I74L) | - |
| **NICU8（M/59）** | NICU8_A43 | NICU | urine | 24/11/2017 | **SRR9316760** | **other** | 1 | 0.25 | 0.12 | 0.25 | 0.12 | 0.06 | 1 | 0.25 | **256** | VF125AL (I74L) | - |
|  | NICU8_A45 | NICU | urine | 25/11/2017 | **SRR9316758** | **other** | 1 | 0.25 | 0.12 | 0.25 | 0.12 | 0.06 | 1 | 0.25 | **256** | VF125AL (I74L) | - |
|  | NICU8_A46 | NICU | urine | 27/11/2017 | **SRR9316767** | **other** | 1 | 0.25 | 0.12 | 0.12 | 0.12 | 0.06 | 1 | 0.25 | **256** | VF125AL (I74L) | - |
|  | NICU8_A47 | NICU | urine | 04/12/2017 | **SRR9316766** | **other** | 1 | 0.25 | 0.12 | 0.25 | 0.06 | 0.06 | 1 | 0.25 | **256** | VF125AL (I74L) | - |
|  | NICU8_A48 | NICU | urine | 06/12/2017 | **SRR9316768** | **other** | 1 | 0.25 | 0.12 | 0.25 | 0.12 | 0.12 | 2 | 0.25 | **256** | VF125AL (I74L) | - |
| **NICU9（M/76）** | NICU9_A50 | NICU | urine | 12/12/2017 | **SRR9316770** | **other** | 1 | 0.12 | 0.06 | 0.12 | 0.12 | 0.06 | 1 | 0.25 | **256** | VF125AL (I74L) | - |
|  | NICU9_A51 | NICU | urine | 16/12/2017 | **SRR9316771** | **other** | 1 | 0.25 | 0.12 | 0.12 | 0.06 | 0.06 | 1 | 0.12 | **256** | VF125AL (I74L) | - |
| **NICU10（F/69）** | NICU10_A58 | NICU | urine | 03/02/2018 | **SRR9316776** | **other** | 1 | 0.25 | 0.12 | 0.25 | 0.12 | 0.12 | 2 | 0.25 | **>256** | VF125AL (I74L) | - |
| **NICU11（F/81）** | NICU11_A77 | NICU | blood | 12/05/2018 | **SRR9316803** | **other** | 1 | 0.5 | 0.12 | 0.25 | 0.12 | 0.12 | 2 | 0.25 | **>256** | VF125AL (I74L) | - |
| **NICU12（M/74）** | NICU12_A79 | NICU | urine | 15/05/2018 | **SRR9316800** | **other** | 1 | 0.12 | 0.06 | 0.12 | 0.12 | 0.06 | 1 | 0.25 | **256** | VF125AL (I74L) | - |
| **NICU13（M/49）** | NICU13_A93 | NICU | urine | 10/08/2018 | **SRR9316812** | **sub-clade1** | 1 | 0.12 | 0.06 | 0.12 | <0.06 | 0.06 | 1 | 0.12 | **256** | VF125AL (I74L) | - |
|  | NICU13_A94 | NICU | urine | 15/08/2018 | **SRR9316817** | **sub-clade1** | 1 | 0.25 | 0.12 | 0.25 | <0.06 | 0.06 | 1 | 0.25 | **>256** | VF125AL (I74L) | - |
| **NICU14（M/61）** | NICU14_A96 | NICU | urine | 27/08/2018 | **SRR9316734** | **other** | 1 | 0.12 | 0.06 | 0.12 | 0.12 | 0.06 | 1 | 0.25 | **256** | VF125AL (I74L) | - |
| **NICU15（M/64）** | NICU15_A100 | NICU | urine | 04/09/2018 | **SRR9316790** | **sub-clade1** | 1 | 0.12 | 0.06 | 0.12 | <0.06 | 0.03 | 0.5 | 0.12 | **256** | VF125AL (I74L) | - |
| **NICU16（M/82）** | NICU16_A101 | NICU | urine | 04/09/2018 | **SRR9316791** | **sub-clade1** | 1 | 0.25 | 0.12 | 0.25 | 0.06 | 0.03 | 1 | 0.12 | **>256** | VF125AL (I74L) | - |
|  | NICU16_A102 | NICU | urine | 07/09/2018 | **SRR9316792** | **sub-clade1** | 0.5 | 0.12 | 0.06 | 0.12 | <0.06 | 0.06 | 0.5 | 0.12 | **256** | VF125AL (I74L) | - |
|  | NICU16_A103 | NICU | urine | 08/09/2018 | **SRR9316793** | **sub-clade1** | 0.5 | 0.25 | 0.12 | 0.25 | 0.12 | 0.06 | 1 | 0.25 | **>256** | VF125AL (I74L) | - |
|  | NICU16_A104 | NICU | urine | 09/09/2018 | **SRR9316794** | **sub-clade1** | 1 | 0.5 | 0.25 | 0.5 | 0.25 | 0.12 | **4** | 0.5 | **>256** | VF125AL (I74L) | - |
| **NSICU1（M/53）** | **NSICU1_A24*** | NSICU | urine | 24/08/2017 | **SRR9316751** | **sub-clade7** | 0.5 | 0.12 | 0.06 | 0.06 | <0.06 | 0.03 | 0.5 | 0.06 | **256** | VF125AL (I74L) | - |
|  | NSICU1_A31 | NSICU | urine | 06/09/2017 | **SRR9316755** | **sub-clade7** | 1 | 0.25 | 0.25 | 0.5 | 0.12 | 0.06 | 2 | 0.5 | **>256** | VF125AL (I74L) | - |
| **SICU1（F/79）** | SICU1_A66 | SICU | urine | 06/04/2018 | **SRR9316780** | **sub-clade5** | 1 | 0.5 | 0.5 | 0.25 | 0.25 | 0.25 | 2 | 0.5 | **>256** | VF125AL (I74L) | - |
|  | SICU1_A67 | SICU | urine | 07/04/2018 | **SRR9316783** | **sub-clade5** | 1 | 0.25 | 0.12 | 0.25 | 0.06 | 0.06 | 1 | 0.25 | **256** | VF125AL (I74L) | - |
|  | SICU1_A68 | SICU | urine | 08/04/2018 | **SRR9316782** | **sub-clade5** | 1 | 0.25 | 0.12 | 0.12 | 0.12 | 0.06 | 1 | 0.25 | **256** | VF125AL (I74L) | - |
|  | SICU1_A70 | SICU | urine | 09/04/2018 | **SRR9316778** | **sub-clade5** | 1 | 0.25 | 0.12 | 0.25 | 0.12 | 0.06 | 1 | 0.25 | **256** | VF125AL (I74L) | - |
|  | SICU1_A71 | SICU | urine | 11/04/2018 | **SRR9316806** | **sub-clade5** | 1 | 0.5 | 0.25 | 0.25 | 0.12 | 0.12 | 2 | 0.25 | **>256** | VF125AL (I74L) | - |
|  | SICU1_A72 | SICU | urine | 12/04/2018 | **SRR9316807** | **sub-clade5** | 1 | 0.5 | 0.25 | 0.25 | 0.25 | 0.12 | 2 | 0.25 | **>256** | VF125AL (I74L) | - |
| **SICU2（M/66）** | SICU2_A89 | SICU | sputum | 25/07/2018 | **SRR9316815** | **other** | 1 | 0.12 | 0.06 | 0.12 | <0.06 | 0.03 | 0.5 | 0.12 | **256** | VF125AL (I74L) | - |
|  | SICU2_A90 | SICU | urine | 29/07/2018 | **SRR9316814** | **sub-clade7** | 1 | 0.25 | 0.12 | 0.25 | 0.12 | 0.06 | 1 | 0.25 | **256** | VF125AL (I74L) | - |
|  | SICU2_A91 | SICU | urine | 30/07/2018 | **SRR9316813** | **other** | 1 | 0.25 | 0.12 | 0.12 | 0.12 | 0.06 | 1 | 0.25 | **256** | VF125AL (I74L) | - |
|  | SICU2_A97 | SICU | urine | 01/09/2018 | **SRR9316735** | **sub-clade7** | 1 | 0.25 | 0.12 | 0.25 | 0.25 | 0.12 | 2 | 0.25 | **256** | VF125AL (I74L) | - |
|  | SICU2_A98 | SICU | urine | 02/09/2018 | **SRR9316736** | **other** | 1 | 0.12 | 0.06 | 0.25 | 0.12 | 0.06 | 1 | 0.25 | **256** | VF125AL (I74L) | - |
| **RI（M/86）** | RI-A112 | RI | urine | 07/11/2018 | **SRR9316720** | **sub-clade2** | 1 | 0.12 | 0.12 | 0.12 | 0.12 | 0.12 | 2 | 0.25 | **>256** | VF125AL (I74L) | - |
|  | RI-A114 | RI | urine | 18/11/2018 | **SRR9316722** | **sub-clade2** | 1 | 0.25 | 0.12 | 0.12 | 0.12 | 0.12 | 2 | 0.5 | **>256** | VF125AL (I74L) | - |
| **Environ-ment** | C12_A109 | RICU | bedrails | 09/03/2018 | **SRR9316725** | **sub-clade6** | 1 | 0.5 | 0.12 | 0.25 | 0.12 | 0.12 | 2 | 0.25 | **256** | VF125AL (I74L) | - |

Note:

*See the previous study (Ref 9)

RICU Respiratory ICU; NICU Neurosciences ICU; NSICU Neurosurgical ICU; SICU Surgical ICU; RI Respiratory infection

AMB: amphotericin B; AFG: anidulafungin; MFG: micafungin; CFG: caspofungin; 5-FC: 5-flucytosine; POS: posaconazole; VRC: voriconazole; ITC: itraconazole; FLU: fluconazole; Boldface for drug-resistant strains. The sequence data of *ERG11* and *FKS1* were submitted to GenBank (serial number MH124608 and MN088094).
